# 4-[2-(Benzyl­sulfan­yl)acet­yl]-3,4-dihydro­quinoxalin-2(1*H*)-one

**DOI:** 10.1107/S1600536811008178

**Published:** 2011-03-12

**Authors:** Waqar Nasir, Munawar Ali Munawar, Sohail Nadeem, Rana Amjad, Ahmad Adnan

**Affiliations:** a Institute of Chemistry, University of the Punjab, Lahore, Pakistan; b Department of Chemistry, GC University, Lahore 54000, Pakistan

## Abstract

In the title compound, C_17_H_16_N_2_O_2_S, the pyrazinone ring is non-planar (r.m.s. deviation = 0.1595 Å), with maximum deviations for the 4-position N atom and the adjacent non-fused-ring C atom of 0.2557 (15) and −0.2118 (16) Å, respectively. The dihedral angle between the benzyl ring and pyrazinone rings is 30.45 (18)°. Inter­molecular N—H⋯O hydrogen-bonding inter­actions forms inversion dimers which lead to eight-membered *R*_2_^2^(8) ring motifs. The dimers are further connected by C—H⋯O inter­actions.

## Related literature

For the biological activity of quinoxalines, see: Ali *et al.* (2000 ▶); Moustafa & Yameda (2001 ▶). For related structures see: Nasir *et al.* (2009) ▶. For graph-set notation, see: Bernstein *et al.* (1995 ▶).
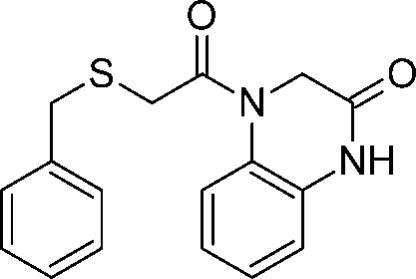


## Experimental

### 

#### Crystal data

C_17_H_16_N_2_O_2_S
*M* *_r_* = 312.38
Orthorhombic, 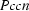

*a* = 13.9502 (8) Å
*b* = 32.2588 (17) Å
*c* = 6.9728 (3) Å
*V* = 3137.9 (3) Å^3^
*Z* = 8
Mo *K*α radiation
μ = 0.22 mm^−1^
*T* = 296 K
0.47 × 0.23 × 0.07 mm

#### Data collection

Bruker Kappa APEXII CCD diffractometer
Absorption correction: multi-scan (*SADABS*; Bruker, 2007 ▶) *T*_min_ = 0.906, *T*_max_ = 0.985
16363 measured reflections
3892 independent reflections
2412 reflections with *I* > 2σ(*I*)
*R*_int_ = 0.044

#### Refinement

*R*[*F* ^2^ > 2σ(*F* ^2^)] = 0.055
*wR*(*F* ^2^) = 0.183
*S* = 1.00
3889 reflections
203 parameters
H atoms treated by a mixture of independent and constrained refinement
Δρ_max_ = 0.27 e Å^−3^
Δρ_min_ = −0.20 e Å^−3^

### 

Data collection: *APEX2* (Bruker, 2007 ▶); cell refinement: *SAINT* (Bruker, 2007 ▶); data reduction: *SAINT*; program(s) used to solve structure: *SHELXS97* (Sheldrick, 2008 ▶); program(s) used to refine structure: *SHELXL97* (Sheldrick, 2008 ▶); molecular graphics: *ORTEP-3 for Windows* (Farrugia, 1997 ▶) and *PLATON* (Spek, 2009 ▶); software used to prepare material for publication: *WinGX* (Farrugia, 1999 ▶) and *PLATON*.

## Supplementary Material

Crystal structure: contains datablocks I, global. DOI: 10.1107/S1600536811008178/hg2799sup1.cif

Structure factors: contains datablocks I. DOI: 10.1107/S1600536811008178/hg2799Isup2.hkl

Additional supplementary materials:  crystallographic information; 3D view; checkCIF report

## Figures and Tables

**Table 1:** Hydrogen-bond geometry (Å, °)

*D*—H⋯*A*	*D*—H	H⋯*A*	*D*⋯*A*	*D*—H⋯*A*
C5—H5⋯O4^i^	0.93	2.60	3.452 (3)	153
C10—H10*B*⋯O4^i^	0.97	2.45	3.202 (3)	134
N2—H1*N*⋯O3^ii^	0.85 (3)	2.02 (3)	2.875 (3)	175 (3)

Symmetry codes: (i) 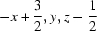; (ii) 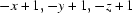.

**Fig. 1.**
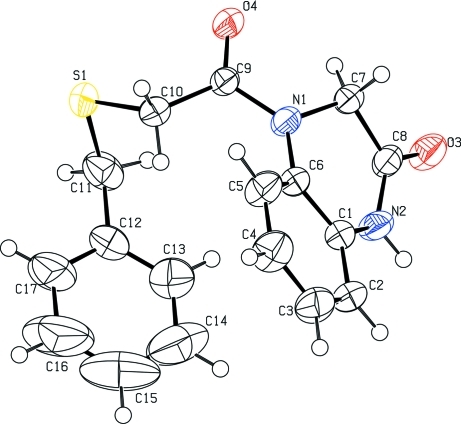
The labelled diagram of structure of (I) with thermal ellipsoids drawn at the 50% probability level.

**Fig. 2.**
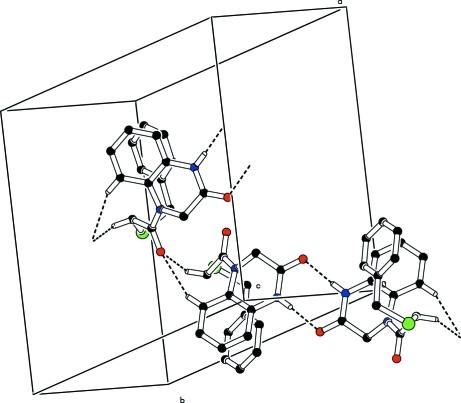
The unit cell packing diagram of (I) showing the hydrogen bondings with dashed lines.

## supplementary crystallographic information

### Comment

Annulated pyrazines like quinoxalinones represents an important class of nitrogen containing heterocyclic compounds possessing wide variety of biological and industrial applications. The synthetic and naturally occurring quinoxalines compounds have been reported to show antibacterial (Ali *et al.*, 2000) and antitumor (Moustafa & Yameda, 2001). In the present project we aimed to synthesize novel quinoxalinone derivatives which may have enhanced biological and pharmaceutical application.

The title compound (I) is in continuation of previously published work on the analoguous structure, 4-[(2,5-dimethylanilino)acetyl]-3,4- dihydroquinoxalin-2(1*H*)-one (II) (Nasir, *et al.*, 2009). The dihedral angle between the aromatic ring (C1/C2/C3/C4/C5/C6) and pyrazinone (C1/C6/N2/C8/C7/N1)is 14.01 (12)°. Unlike (II) no intramolecular hydrogen bonding have been observed in (I). The N—H···O type intermolecular hydrogen bonding developed from the cyclic amido functional group forms the inversion dimers and produce eight membered ring motif R_2_^2^(8) (Bernstein *et al.*, 1995). Another C—H···O type hydrogen bonding interaction connects these dimers to another molecule Fig. 2. The benzyl ring (C12/C13/C14/C15/C16/C17) is oriented at dihedral angle of 14.01 (12)° and 30.45 (18)° with respect to aromatic and pyrazinone rings.

### Experimental

To a suspension of 4-(chloroacetyl)-3,4-dihydroquinoxalin-2(1*H*)-2-one (2.0 g, 8.9 mmoles) in absolute ethanol (60 mL)  fine powdered sodium bicarbonate (1.5 g, 17.8 mmole) was added along with phenylmethanethiol (1.1 mL, 9.0 mmoles). The reaction mixture was heated under reflux for 8-10 h, the progress of the reaction was monitored by TLC (chloroform:ethyl acetate, 7:3 v/v). The reaction mixture was concentrated to half of the original volume under reduced pressure and the precipitate of the product which formed on cooling was filtered, washed with cold ethanol and recrystallized in ethanol.

### Refinement

All the C—H and N—H H-atoms were positioned with idealized geometry with C—H = 0.93 Å for aromatic, with C—H = 0.97 Å for methylene and with N—H = 0.85 (3)Å for amido NH and were refined using a riding model with *U*_iso_(H) = 1.2 *U*_eq_(C & N). The reflection 1 1 0, 1 3 0 and 0 2 0 were omitted in final refinement as these were obscured by the beam stop.

### Crystal data

**Table:**

C_17_H_16_N_2_O_2_S	*F*(000) = 1312
*M**_r_* = 312.38	*D*_x_ = 1.322 Mg m^−^^3^
Orthorhombic, *P**c**c**n*	Mo *K*α radiation, λ = 0.71073 Å
Hall symbol: -P 2ab 2ac	Cell parameters from 2915 reflections
*a* = 13.9502 (8) Å	θ = 3.2–23.1°
*b* = 32.2588 (17) Å	µ = 0.22 mm^−^^1^
*c* = 6.9728 (3) Å	*T* = 296 K
*V* = 3137.9 (3) Å^3^	Plate, colorless
*Z* = 8	0.47 × 0.23 × 0.07 mm

### Data collection

**Table:**

Bruker Kappa APEXII CCD diffractometer	3892 independent reflections
Radiation source: fine-focus sealed tube	2412 reflections with *I* > 2σ(*I*)
graphite	*R*_int_ = 0.044
φ and ω scans	θ_max_ = 28.3°, θ_min_ = 1.3°
Absorption correction: multi-scan (*SADABS*; Bruker, 2007)	*h* = −18→17
*T*_min_ = 0.906, *T*_max_ = 0.985	*k* = −23→43
16363 measured reflections	*l* = −9→9

### Refinement

**Table:**

Refinement on *F*^2^	Primary atom site location: structure-invariant direct methods
Least-squares matrix: full	Secondary atom site location: difference Fourier map
*R*[*F*^2^ > 2σ(*F*^2^)] = 0.055	Hydrogen site location: inferred from neighbouring sites
*wR*(*F*^2^) = 0.183	H atoms treated by a mixture of independent and constrained refinement
*S* = 1.00	*w* = 1/[σ^2^(*F*_o_^2^) + (0.108*P*)^2^ + 0.1052*P*] where *P* = (*F*_o_^2^ + 2*F*_c_^2^)/3
3889 reflections	(Δ/σ)_max_ = 0.001
203 parameters	Δρ_max_ = 0.27 e Å^−^^3^
0 restraints	Δρ_min_ = −0.20 e Å^−^^3^

### Special details

**Table:**

Geometry. All s.u.'s (except the s.u. in the dihedral angle between two l.s. planes) are estimated using the full covariance matrix. The cell s.u.'s are taken into account individually in the estimation of s.u.'s in distances, angles and torsion angles; correlations between s.u.'s in cell parameters are only used when they are defined by crystal symmetry. An approximate (isotropic) treatment of cell s.u.'s is used for estimating s.u.'s involving l.s. planes.
Refinement. Refinement of F^2^ against ALL reflections. The weighted R-factor wR and goodness of fit S are based on F^2^, conventional R-factors R are based on F, with F set to zero for negative F^2^. The threshold expression of F^2^ > 2σ(F^2^) is used only for calculating R-factors(gt) etc. and is not relevant to the choice of reflections for refinement. R-factors based on F^2^ are statistically about twice as large as those based on F, and R- factors based on ALL data will be even larger.

### Fractional atomic coordinates and isotropic or equivalent isotropic displacement parameters (Å^2^)

**Table:**

	*x*	*y*	*z*	*U*_iso_*/*U*_eq_	
S1	0.67947 (5)	0.701430 (18)	0.13947 (11)	0.0539 (3)	
O4	0.77529 (11)	0.61610 (5)	0.1173 (2)	0.0439 (4)	
N1	0.63517 (13)	0.58234 (5)	0.0968 (3)	0.0364 (4)	
O3	0.63199 (12)	0.50899 (5)	0.4824 (2)	0.0530 (5)	
C1	0.47960 (15)	0.55216 (6)	0.1309 (3)	0.0377 (5)	
C9	0.69061 (15)	0.61743 (6)	0.0743 (3)	0.0331 (5)	
C8	0.60499 (16)	0.52927 (7)	0.3446 (3)	0.0399 (5)	
C7	0.67595 (15)	0.54813 (7)	0.2064 (3)	0.0400 (5)	
H7A	0.7311	0.5581	0.2775	0.048*	
H7B	0.6980	0.5269	0.1184	0.048*	
N2	0.51165 (14)	0.53532 (6)	0.3046 (3)	0.0442 (5)	
C10	0.64388 (16)	0.65674 (7)	0.0037 (3)	0.0394 (5)	
H10A	0.5748	0.6537	0.0112	0.047*	
H10B	0.6607	0.6610	−0.1299	0.047*	
C5	0.51272 (19)	0.58963 (8)	−0.1586 (4)	0.0490 (6)	
H5	0.5557	0.6032	−0.2391	0.059*	
C12	0.50134 (19)	0.69134 (8)	0.3071 (4)	0.0514 (6)	
C6	0.54236 (15)	0.57563 (6)	0.0199 (3)	0.0364 (5)	
C3	0.35632 (19)	0.56161 (9)	−0.1015 (4)	0.0581 (7)	
H3	0.2931	0.5579	−0.1402	0.070*	
C2	0.38689 (17)	0.54535 (7)	0.0697 (4)	0.0477 (6)	
H2	0.3451	0.5297	0.1445	0.057*	
C11	0.6058 (2)	0.69568 (9)	0.3512 (4)	0.0577 (7)	
H11A	0.6269	0.6714	0.4217	0.069*	
H11B	0.6150	0.7197	0.4332	0.069*	
C4	0.4191 (2)	0.58341 (8)	−0.2165 (4)	0.0592 (7)	
H4	0.3983	0.5940	−0.3334	0.071*	
C13	0.4534 (3)	0.65588 (11)	0.3446 (5)	0.0776 (9)	
H13	0.4853	0.6339	0.4025	0.093*	
C16	0.3583 (3)	0.7183 (2)	0.1691 (9)	0.156 (3)	
H16	0.3267	0.7396	0.1054	0.188*	
C14	0.3569 (3)	0.65185 (15)	0.2976 (7)	0.1123 (16)	
H14	0.3240	0.6275	0.3254	0.135*	
C17	0.4533 (2)	0.72278 (13)	0.2194 (7)	0.1095 (15)	
H17	0.4849	0.7475	0.1932	0.131*	
C15	0.3119 (3)	0.6836 (2)	0.2115 (7)	0.133 (2)	
H15	0.2473	0.6812	0.1811	0.160*	
H1N	0.471 (2)	0.5207 (9)	0.367 (4)	0.060 (8)*	

### Atomic displacement parameters (Å^2^)

**Table:**

	*U*^11^	*U*^22^	*U*^33^	*U*^12^	*U*^13^	*U*^23^
S1	0.0418 (4)	0.0307 (3)	0.0892 (6)	−0.0038 (2)	0.0018 (3)	−0.0011 (3)
O4	0.0330 (9)	0.0376 (8)	0.0611 (10)	−0.0068 (7)	−0.0027 (7)	0.0045 (7)
N1	0.0310 (10)	0.0358 (10)	0.0424 (10)	−0.0056 (8)	−0.0005 (8)	0.0066 (8)
O3	0.0451 (10)	0.0577 (11)	0.0561 (11)	−0.0119 (8)	−0.0068 (8)	0.0193 (9)
C1	0.0321 (11)	0.0339 (11)	0.0473 (13)	−0.0016 (9)	0.0012 (10)	0.0006 (10)
C9	0.0322 (11)	0.0316 (10)	0.0356 (11)	−0.0031 (9)	0.0043 (9)	−0.0004 (9)
C8	0.0380 (12)	0.0360 (11)	0.0456 (13)	−0.0075 (10)	0.0005 (10)	0.0048 (10)
C7	0.0315 (11)	0.0358 (11)	0.0527 (13)	−0.0028 (9)	0.0015 (10)	0.0092 (10)
N2	0.0336 (11)	0.0494 (11)	0.0495 (12)	−0.0061 (9)	0.0057 (9)	0.0140 (10)
C10	0.0355 (12)	0.0381 (12)	0.0447 (12)	−0.0009 (10)	0.0063 (10)	0.0042 (10)
C5	0.0462 (15)	0.0544 (15)	0.0464 (14)	−0.0109 (12)	−0.0015 (11)	0.0093 (11)
C12	0.0476 (15)	0.0572 (15)	0.0495 (14)	0.0108 (12)	0.0027 (11)	−0.0060 (12)
C6	0.0302 (11)	0.0332 (10)	0.0459 (12)	−0.0037 (9)	−0.0009 (9)	−0.0002 (9)
C3	0.0391 (13)	0.0587 (16)	0.0765 (19)	−0.0109 (13)	−0.0155 (13)	−0.0009 (14)
C2	0.0338 (12)	0.0452 (13)	0.0642 (16)	−0.0086 (11)	0.0019 (11)	0.0022 (12)
C11	0.0540 (17)	0.0592 (16)	0.0599 (16)	0.0099 (13)	−0.0070 (13)	−0.0154 (13)
C4	0.0557 (17)	0.0663 (17)	0.0558 (16)	−0.0097 (14)	−0.0181 (13)	0.0063 (13)
C13	0.068 (2)	0.075 (2)	0.090 (2)	−0.0013 (18)	0.0167 (18)	−0.0014 (18)
C16	0.062 (3)	0.211 (6)	0.197 (6)	0.044 (3)	0.011 (3)	0.093 (5)
C14	0.076 (3)	0.135 (4)	0.126 (4)	−0.044 (3)	0.031 (3)	−0.041 (3)
C17	0.053 (2)	0.099 (3)	0.176 (4)	0.028 (2)	0.007 (2)	0.054 (3)
C15	0.050 (2)	0.241 (7)	0.109 (4)	0.011 (3)	−0.010 (2)	−0.007 (4)

### Geometric parameters (Å, °)

**Table:**

S1—C10	1.795 (2)	C5—H5	0.9300
S1—C11	1.808 (3)	C12—C13	1.351 (4)
O4—C9	1.219 (3)	C12—C17	1.361 (4)
N1—C9	1.380 (3)	C12—C11	1.496 (4)
N1—C6	1.418 (3)	C3—C2	1.372 (4)
N1—C7	1.458 (3)	C3—C4	1.380 (4)
O3—C8	1.222 (3)	C3—H3	0.9300
C1—C2	1.380 (3)	C2—H2	0.9300
C1—C6	1.393 (3)	C11—H11A	0.9700
C1—N2	1.400 (3)	C11—H11B	0.9700
C9—C10	1.508 (3)	C4—H4	0.9300
C8—N2	1.346 (3)	C13—C14	1.392 (5)
C8—C7	1.509 (3)	C13—H13	0.9300
C7—H7A	0.9700	C16—C15	1.327 (8)
C7—H7B	0.9700	C16—C17	1.379 (6)
N2—H1N	0.85 (3)	C16—H16	0.9300
C10—H10A	0.9700	C14—C15	1.342 (7)
C10—H10B	0.9700	C14—H14	0.9300
C5—C4	1.382 (4)	C17—H17	0.9300
C5—C6	1.387 (3)	C15—H15	0.9300
			
C10—S1—C11	101.02 (12)	C5—C6—C1	119.2 (2)
C9—N1—C6	126.45 (18)	C5—C6—N1	124.2 (2)
C9—N1—C7	117.51 (17)	C1—C6—N1	116.55 (19)
C6—N1—C7	116.03 (17)	C2—C3—C4	120.2 (2)
C2—C1—C6	120.3 (2)	C2—C3—H3	119.9
C2—C1—N2	120.3 (2)	C4—C3—H3	119.9
C6—C1—N2	119.4 (2)	C3—C2—C1	120.0 (2)
O4—C9—N1	119.08 (19)	C3—C2—H2	120.0
O4—C9—C10	121.91 (19)	C1—C2—H2	120.0
N1—C9—C10	118.98 (19)	C12—C11—S1	113.29 (19)
O3—C8—N2	122.6 (2)	C12—C11—H11A	108.9
O3—C8—C7	121.0 (2)	S1—C11—H11A	108.9
N2—C8—C7	116.3 (2)	C12—C11—H11B	108.9
N1—C7—C8	112.57 (18)	S1—C11—H11B	108.9
N1—C7—H7A	109.1	H11A—C11—H11B	107.7
C8—C7—H7A	109.1	C3—C4—C5	120.3 (3)
N1—C7—H7B	109.1	C3—C4—H4	119.9
C8—C7—H7B	109.1	C5—C4—H4	119.9
H7A—C7—H7B	107.8	C12—C13—C14	120.8 (4)
C8—N2—C1	123.0 (2)	C12—C13—H13	119.6
C8—N2—H1N	117.0 (19)	C14—C13—H13	119.6
C1—N2—H1N	116.3 (19)	C15—C16—C17	120.0 (5)
C9—C10—S1	112.54 (16)	C15—C16—H16	120.0
C9—C10—H10A	109.1	C17—C16—H16	120.0
S1—C10—H10A	109.1	C15—C14—C13	119.1 (4)
C9—C10—H10B	109.1	C15—C14—H14	120.4
S1—C10—H10B	109.1	C13—C14—H14	120.4
H10A—C10—H10B	107.8	C12—C17—C16	120.7 (4)
C4—C5—C6	119.8 (2)	C12—C17—H17	119.7
C4—C5—H5	120.1	C16—C17—H17	119.7
C6—C5—H5	120.1	C16—C15—C14	121.1 (4)
C13—C12—C17	118.3 (3)	C16—C15—H15	119.5
C13—C12—C11	121.4 (3)	C14—C15—H15	119.5
C17—C12—C11	120.2 (3)		
			
C6—N1—C9—O4	−166.4 (2)	C9—N1—C6—C5	36.4 (3)
C7—N1—C9—O4	12.1 (3)	C7—N1—C6—C5	−142.1 (2)
C6—N1—C9—C10	15.7 (3)	C9—N1—C6—C1	−145.9 (2)
C7—N1—C9—C10	−165.86 (19)	C7—N1—C6—C1	35.6 (3)
C9—N1—C7—C8	136.4 (2)	C4—C3—C2—C1	2.5 (4)
C6—N1—C7—C8	−44.9 (3)	C6—C1—C2—C3	0.2 (4)
O3—C8—C7—N1	−159.2 (2)	N2—C1—C2—C3	−179.4 (2)
N2—C8—C7—N1	22.2 (3)	C13—C12—C11—S1	114.1 (3)
O3—C8—N2—C1	−168.6 (2)	C17—C12—C11—S1	−62.9 (4)
C7—C8—N2—C1	10.0 (3)	C10—S1—C11—C12	−54.1 (2)
C2—C1—N2—C8	158.4 (2)	C2—C3—C4—C5	−1.1 (4)
C6—C1—N2—C8	−21.2 (3)	C6—C5—C4—C3	−3.1 (4)
O4—C9—C10—S1	−43.2 (3)	C17—C12—C13—C14	−1.1 (5)
N1—C9—C10—S1	134.71 (18)	C11—C12—C13—C14	−178.1 (3)
C11—S1—C10—C9	−78.50 (18)	C12—C13—C14—C15	1.0 (6)
C4—C5—C6—C1	5.8 (4)	C13—C12—C17—C16	−0.4 (6)
C4—C5—C6—N1	−176.6 (2)	C11—C12—C17—C16	176.6 (4)
C2—C1—C6—C5	−4.4 (3)	C15—C16—C17—C12	2.1 (9)
N2—C1—C6—C5	175.2 (2)	C17—C16—C15—C14	−2.3 (10)
C2—C1—C6—N1	177.8 (2)	C13—C14—C15—C16	0.7 (8)
N2—C1—C6—N1	−2.6 (3)		

### Hydrogen-bond geometry (Å, °)

**Table:**

*D*—H···*A*	*D*—H	H···*A*	*D*···*A*	*D*—H···*A*
C5—H5···O4^i^	0.93	2.60	3.452 (3)	153
C10—H10B···O4^i^	0.97	2.45	3.202 (3)	134
N2—H1N···O3^ii^	0.85 (3)	2.02 (3)	2.875 (3)	175 (3)

Symmetry codes: (i) −*x*+3/2, *y*, *z*−1/2; (ii) −*x*+1, −*y*+1, −*z*+1.
